# Responses of maize hybrids with contrasting maturity to planting date in Northeast China

**DOI:** 10.1038/s41598-021-95328-5

**Published:** 2021-08-04

**Authors:** Fulai Ke, Xinglin Ma

**Affiliations:** Institute of Crop Science, Chinese Academy of Agricultural Sciences (CAAS), Beijing, 100081 China

**Keywords:** Genetics, Plant sciences

## Abstract

Maize sowing in semi-humid region of Jilin province is often delayed beyond the optimum planting time window because of soil water stress typically occurring before or during the planting season. Research was conducted at Lishu city, in Jilin province from 2009 through 2010 to determine the responses of maize hybrids with contrasting maturity to planting date. Three popular hybrids with contrasting different maturity, short-season hybrid Jidan27, mid-season hybrid Xianyu335, and full-season hybrid Zhengdan958 were planted in early May and mid-May and thinned to populations of 67,500 plants ha^−1^. The results showed that durations from emergence to silking stage for all the hybrids consistently shortened as the planting delayed, but interaction effects of hybrids, planting date and year existed for the duration of silking to physiological maturity stage. The longer maturity hybrid usually had grain yield advantage over earlier maturity hybrid when planting at early May, but the earlier maturity hybrid often showed a greater yield than longer maturity hybrid when planting was delayed. The highest yield occurred at the treatment combination of the mid-season hybrid and delayed planting date, and the shorter season hybrid typically showed stable and higher grain yield across planting dates. Changes in grain volume per unit of land area among hybrids and planting date treatment combination were consistent with the changes in grain yields, indicating that the yield is determined usually by the sink capacity. Further research is needed to evaluate the effects of hybrids maturity and planting date on maize under different planting densities.

## Introduction

Maize in Northeast China (includes provinces of Heilongjiang, Jilin, Liaoning, and the northeastern part of Inner Mongolia) accounts for approximately 38.8% of total area and 43.4% of total production of the whole country^1^. Maize there, usually called “spring corn”, is often sowed in late April to early May and is mainly rainfed crop^2,3^. Timely maize planting is critical for maximizing grain yield in Northeast China^4^. However, if drought or waterlogging occurs during the sowing season, the planting date will be delayed. In fact, drought is a frequent occurrence in Northeast China during the maize sowing period^5–10^, waterlogging also occasionally occurred during this period^11,12^. When planting is delayed, the cumulative time and thermal requirement for regionally adapted maize hybrids to reach black layer shortens^13^. In this case, it is necessary considering whether to switch to earlier-maturity hybrids to ensure that physiological grain maturity occurs before a killing fall frost and stable yield can be obtained. Therefore, it is necessary to answer the question of how maize hybrids with contrasting maturity respond to delayed sowing dates.

There are many researches on the effect of sowing date on the growth and yield of maize in China^14–22^. Lv et al.^23^ and Lu et al.^24^ reported that different sowing dates changed the configuration of climatic elements such as light, heat and water during the growth stage of maize. The results of Liu et al.^18^ and Li et al.^14^ showed that sowing date affected the growth and development progress of maize, and appropriate early sowing could increase the effective accumulative temperature, prolong the growth period of maize and increase dry matter accumulation, which was conducive to the realization of high yield of maize. In Western Kansas, Norwood^25^ found dryland corn yield for the early May planting date were always higher than those for the mid-April planting date. In north-central and northeast Kansas, Staggenborg et al.^26^ found that planting in early April or early May produced similar yields. In comparison, Lauer et al.^27^ found that corn grain yield of both full- and shorter-season hybrids over 12 site-years in southern Wisconsin was maximized with planting dates of 1–7 May, and that yield was reduced by 0.5 to 1.1% for each 1-d delay in planting between 8 and 22 May. When planting was delayed from 22 May to 5 June, yield continued to decline by 1.3–1.9% d^–1^.

Previous studies have shown that there is a significant positive effect between yield and hybrid maturity^28^. In recent years, some researchers in northeast China began to pay attention to the yield performance between hybrids with contrasting maturity. Shi et al.^29^ reported that at present, longer-maturity hybrids have been dominant in corn production in Liaoning province. It was reported that the yield of the shorter-season hybrids was not lower than that of full-season hybrids in Liaoning province^30,31^ and^32^. Traditionally, in Northeast China, full-season or ultra-full-season hybrids have been used by producers, even when delayed planting operations occurred. Accordingly, researches in this region only focused on risk of late-maturity maize hybrids under extreme climate conditions^8^ and difference in maize yield between mid- and late-maturing hybrids^33^. Incredibly, there is little research on the planting date- maize hybrid maturity effects.

Maize production in the Midsouth Jilin province typically entails planting hybrid that requires 120–130 days from emergence to physiological maturity^3,34^. Planting dates are currently in late April or early May^3,34^. As mentioned above, this area characterized by typically low or occasionally high moisture content in the soil before or during the planting season. When the area encounters these soil water stress conditions, planting date have to be delayed. Then, the grain-filling period of full-season hybrids would be more affected by the unfavorable environmental conditions during reproductive growth stage than that of short or mid-season hybrids. Short-season hybrids would place reproductive stage under more favorable environmental conditions than their long-season counterparts as Lauer et al.^27^ observed in U. S. Corn Belt. Therefore, short-season maize hybrids (≈ 105 days from emergence to maturity) would seemingly have potential for delayed planting resulted from spring drought or excessive rainfall in the Midsouth Jilin. However, since short-season maize hybrids are primarily grown in the northern part of Jilin and Heilongjiang province, there is little research on the adaptability of short-season hybrids in this area. The objectives of this research were to (i) evaluate the potential of short-season maize hybrids for delayed planting in the Midsouth Jilin, and (ii) determine if yield of short-season maize in Midsouth Jilin was similar to full-season hybrids currently being produced in the area, and (iii) provide information for all the Northeast China (where different heat units among different regions existed, but all of the regions have their own full-season and short-season hybrids) on the relationship between maize yield and hybrid maturity-planting date interaction.

## Results

### Climatic conditions

In general, the maximum, minimum, and average temperatures of 2009 were very similar to those of 2010 for the entire maize growing period, while the precipitation in 2010 was much higher than in 2009 (Table 1). Furthermore, the precipitation in 2010 was significantly higher than in 2009, before, after and during the maize flowering. The cumulative precipitation before, after and during maize flowering in 2010 was 219, 284 and 128 mm, which were 1.4, 4.5, and 3.4 times of that in 2009 (Table 1).

**Table 1:** Mean monthly minimum (min. temp.), maximum (max. temp.), and average temperature (ave.temp.) and precipitation from May through September at experimental site in 2009 and 2010.

2009					2010				
Time period	Max. temp. (℃)	Min. temp. (℃)	Ave. temp. (℃)	Precipitation (mm)	Time period	Max. temp. (℃)	Min. temp. (℃)	Ave. temp. (℃)	Precipitation (mm)
May	24.6	11.9	18.3	40.5	May	21.9	11.3	16.6	82.3
June	24.7	15.0	19.9	69.6	June	29.7	17.7	23.7	16.1
1–17 July	27.2	17.8	22.5	41.4	1–15 July	27.5	18.2	22.9	120.9
18–29 July^a^	28.4	18.1	23.3	37.2	16–27 July^a^	28.0	20.5	24.3	128.3
30–31 July	28.3	17.8	23.1	2.1	28–31 July	27.9	20.6	24.3	30.2
Aug	29.3	17.4	23.4	57.4	Aug	26.4	17.9	22.2	217.2
Sept	23.2	9.9	16.6	3.9	Sept	22.9	11.2	17.1	36.6

^a^Flowering period.

### Days from emergence to silking, silking to physiological maturity, and emergence to physiological maturity, and number of leaves per plant

Days from emergence to silking (DES) was significantly decreased by delayed planting except for mid-season hybrid XY335 in 2009 (Table 2). In both years, short-season hybrid JD27 had significantly lower DES than the mid- and full-season hybrids at each planting date treatment. Difference for DES between mid- and full-season hybrids at early May planting treatment (D1) was not significant in both years. At mid-May planting treatment (D2), the mid-season hybrid XY335 had significant higher DES than the full-season hybrid ZD958 in 2009, and had significant lower DES than ZD958 in 2010.

**Table 2:** Effects of planting date and hybrid maturity on days from emergence to silking, silking to physiological maturity, and emergence to physiological maturity, and number of leaves per plant of dryland corn at Lishu city, Northeast China during 2009 and 2010 growing seasons.

	2009			2010		
	JD27	XY335	ZD958	JD27	XY335	ZD958
Planting date	Days from emergence to silking (days)					
D1^c^	66.3B^a^	70.3A	70.3A	61.3B	65.3A	66.3A
D2^d^	64.3C	69.3A	67.7B	56.3Bc	61.7Ab	63.0Aa
Difference	2.0**	1.0NS^b^	2.7**	5.0**	3.7**	3.3**
Planting date	Days from silking to physiological maturity (days)					
D1	44.0C	53.0B	58.0A	46.0C	52.0B	58.0A
D2	43.0C	51.0B	59.7A	46.0C	55.0B	61.3A
Difference	1.0NS	2.0*	1.7*	0.0NS	3.0**	3.3**
Planting date	Days from emergence to physiological maturity (days)					
D1	110.3C	123.3B	128.3A	107.3C	117.3B	124.3A
D2	107.3C	120.3B	127.3A	102.3C	116.7B	124.3A
Difference	3.0**	3.0**	1.0NS	5.0**	0.7NS	0NS
Planting date	Number of leaves per plant					
D1	19.3B	20.5A	20.3A	19.2B	20.6A	20.6A
D2	19.5B	20.5A	20.8A	19.6b	19.9ab	20.5a
Difference	0.2NS	0NS	0.5*	0.5NS	0.7NS	0.1NS

^a^Means within a row followed by a different lower-case letter differ at *P* < 0.05. Means within a row followed by a different upper-case letter differ at *P* < 0.01.

^b^NS, not significant.

^c^D1 represents the planting date of maize in early May. The planting dates for 2009 and 2010 were May 2 and May 4, respectively.

^d^D2 represents the planting date of maize in mid-May. The planting dates for 2009 and 2010 were May 12 and May 14, respectively.

*Significant at the 0.05 level.

**Significant at the 0.01 level.

Days from silking to physiological maturity (DSPM) significantly increased with hybrid maturity (Table 2). In both seasons, there were no significant effects of planting date on DSPM for JD27; In 2009, DSPM of D1 significantly higher than that of D2 for XY335; In 2010, DSPM of D1 significantly lower than that of D2 for XY335; In both seasons, DSPM of D1 significantly lower than those of D2 for ZD958.

Days from emergence to physiological maturity (DEPM) significantly increased with hybrid maturity (Table 2). In both seasons, D2 resulted in statistically lower DEPM than D1 for JD27; In 2009, D1 showed higher DEPM than D2 for XY335; In 2010, the difference between planting date treatments for DEPM for XY335 was not significant; In both seasons, no significant difference was found between the two planting date treatment for ZD958.

The shorter season hybrid JD27 had a significantly lower number of leaves per plant (NLPP) than the longer season hybrids (i.e. mid- and full- season hybrid) (Table 2). Difference of NLPP between mid- and full-season hybrids was not observed. No difference of NLPP between planting dates was found, except for ZD958 in 2009 which had more leaves at D2 than D1.

### Grain yield, kernel water content at harvest stage, and kernel density

Significant interactions between hybrid maturity and planting date for grain yield were found both in 2009 and 2010 seasons (Table 3). In both seasons, D1 showed significantly (*P* < 0.01) lower grain yield than D2 both for JD27 and XY335. By contrast, grain yield of D1 was significantly (*P* < 0.01) higher than that of D2 for ZD958. In contrast to grain yield across hybrids, with similar grain yield between XY335 and ZD958 at D1 in 2009, they produced significantly greater grain than JD27. With similar grain yield between JD27 and XY335 at D2 in 2009, they showed significantly higher grain yield than ZD958; In 2010, grain yield significantly increased with hybrid maturity at D1. XY335 and ZD958 exhibited the highest and the least grain yield at D2. Delayed planting significantly affected grain yield for all three hybrids.

**Table 3:** Effects of planting date and hybrid maturity on grain yield, kernel water content at harvest stage and kernel density of dryland corn at Lishu city, Northeast China during 2009 and 2010 growing seasons.

	2009			2010		
	JD27	XY335	ZD958	JD27	XY335	ZD958
Planting date	Grain yield kg ha^−1^					
D1^c^	10064b^a^	10549a	10409a	7986C	9022B	10718A
D2^d^	10889A	11176A	8560B	8686B	10061A	7700C
Difference	825**	627**	1849**	700**	1039**	3018**
Planting date	Kernel water content at harvest stage %					
D1	15.1C	20.8B	28.8A	14.2C	18.4B	24.9A
D2	16.6C	22.9B	31.9A	15.4C	21.0B	27.9A
Difference	1.5**	2.1**	3.1**	1.2**	2.6**	3.0**
Planting date	Kernel density g cm^−3^					
D1	1.30b	1.33a	1.30b	1.30ab	1.27b	1.32a
D2	1.31a	1.29a	1.28a	1.27b	1.31a	1.28ab
Difference	0.02NS^b^	0.04*	0.02NS	0.03NS	0.04NS	0.04*

^a^Means within a row followed by a different lower-case letter differ at *P* < 0.05. Means within a row followed by a different upper-case letter differ at *P* < 0.01.

^b^NS, not significant.

^c^D1 represents the planting date of maize in early May. The planting dates for 2009 and 2010 were May 2 and May 4, respectively.

^d^D2 represents the planting date of maize in mid-May. The planting dates for 2009 and 2010 were May 12 and May 14, respectively.

^*^Significant at the 0.05 level.

^**^Significant at the 0.01 level.

As expected, kernel water content at harvest stage (KWCHS) significantly (*P* < 0.01) increased with hybrid maturity (Table 3). This would be associated with unfavorable profitability of maize production resulted from drying costs. In both seasons, three hybrids showed significant differences with the order of KWCHS: ZD958>XY335>JD27.

Significant interactions between hybrid maturity and planting date for kernel density were found both in 2009 and 2010 growing seasons (Table 3). In 2009, with similar kernel density among hybrids at D2, XY335 had significantly (*P* < 0.05) higher kernel density than JD27 and ZD958 at D1. In 2010, significant differences were found between XY335 and ZD958 at D1, and between XY335 and JD27 at D2. Considering the difference between planting date treatments, the kernel density for XY335 in 2009 and ZD958 in 2010 at D1were significantly higher than D2. It indicated that delayed planting exhibited a trend of decrease in kernel density.

### Aboveground biomass at harvest stage, and harvest index

In both seasons, significant interactions between hybrid maturity and planting date for aboveground biomass at harvest stage (ABHS) were observed (Table 4). Given the difference between planting date treatments, ABHS of XY335 and ZD958 exhibited significant differences in 2009. In contrast to ABHS across hybrids, in 2009, ABHS of XY335 was significantly higher than the others, and ABHS of ZD958 was significantly higher than JD27 at D1; At D2, ABHS of XY335 was significantly higher than JD27, and ABHS of JD27 significantly higher than ZD958. In 2010, ZD 958 produced significantly greater ABHS than the others, and no difference in ABHS between JD27 and XY335 was found at D1; At D2, JD27 had significantly lower ABHS than the others.

**Table 4:** Effects of planting date and hybrid maturity on aboveground biomass at harvest stage, and harvest index of dryland corn at Lishu city, Northeast China during 2009 and 2010 growing seasons.

	2009			2010		
	JD27	XY335	ZD958	JD27	XY335	ZD958
Planting date	Aboveground biomass at harvest stage kg ha^-1^					
D1^c^	16487Bb^a^	19054A	17273Ba	16323b	17158b	19496a
D2^d^	16904B	19859A	15975C	15289b	18774a	17724a
Difference	418NS^b^	805**	1298**	1034NS	1616NS	1772NS
Planting date	Harvest index %					
D1	61.0A	55.4C	60.3B	49.2b	52.6ab	55.1a
D2	64.4A	56.3B	53.6C	56.8A	53.6A	43.9B
Difference	3.4**	0.9**	6.7**	7.6*	1.0NS	11.2**

^a^Means within a row followed by a different lower-case letter differ at *P* < 0.05. Means within a row followed by a different upper-case letter differ at *P* < 0.01.

^b^NS, not significant.

^c^D1 represents the planting date of maize in early May. The planting dates for 2009 and 2010 were May 2 and May 4, respectively.

^d^D2 represents the planting date of maize in mid-May. The planting dates for 2009 and 2010 were May 12 and May 14, respectively.

^*^Significant at the 0.05 level.

^**^Significant at the 0.01 level.

Significant interactions between hybrid maturity and planting date for harvest index (HI) were found in both seasons (Table 4). In 2009, D1 had significantly lower HI than D2 both for JD27 and XY335; In 2010, D1 also had significantly lower HI than D2 for XY335; In both seasons, D1 had significantly greater HI than D2 for ZD958. In contrast to HI across hybrids, JD27 exhibited highest HI at both planting date treatments in 2019. In 2010, ZD958 and JD27 had the highest HI at D1 and D2.

The results suggested that both aboveground biomass at harvest and harvest index contributed to grain yield differences between treatments of the experiment.

### Yield components

There were no significant effects of hybrid maturity and planting date on number of ears m^−^^2^ in 2009. In 2010, significant interaction between hybrid maturity and planting date for number of ears m^−2^ was found. At D2, XY335 exhibited significantly lower number of ears m^−2^ than JD27 and ZD958. Considering planting date difference, D2 exhibited significantly lower number of ears m^−2^ than D1 for XY335 (Table 5).

**Table 5:** Effects of planting date and hybrid maturity on number of ears m^-2^, grain weight per ear, number of grains per ear, and 100-grain weight of dryland corn at Lishu city, Northeast China during 2009 and 2010 growing seasons.

	2009			2010		
	JD27	XY335	ZD958	JD27	XY335	ZD958
Planting date	Number of ears m^−2^					
D1^c^	6.68a^a^	6.47a	6.47a	6.68a	6.75a	6.75a
D2^d^	6.54a	6.47a	6.47a	6.68A	6.40B	6.75A
Difference	0.14NS^b^	0NS	0NS	0NS	0.35**	0.00NS
Planting date	Grain weight per ear g ear^−1^					
D1	151B	163A	161A	120C	134B	159A
D2	167A	173A	132B	130B	157A	114C
Difference	16**	10*	29**	10**	23**	45**
Planting date	Number of grains per ear					
D1	418b	508a	498a	315B	431A	449A
D2	451ABb	530Aa	435Bb	401AB	446A	337B
Difference	33NS	22NS	63*	86*	15NS	112**
Planting date	100-grain weight g					
D1	36.4a	32.1a	32.3a	38.0a	31.0b	36.1ab
D2	37.0a	32.6b	30.7b	32.6a	35.3a	34.0a
Difference	0.6NS	0.5NS	1.6NS	5.4NS	4.3NS	2.1NS

^a^Means within a row followed by a different lower-case letter differ at *P* < 0.05. Means within a row followed by a different upper-case letter differ at *P* < 0.01.

^b^NS, not significant.

^c^D1 represents the planting date of maize in early May. The planting dates for 2009 and 2010 were May 2 and May 4, respectively.

^d^D2 represents the planting date of maize in mid-May. The planting dates for 2009 and 2010 were May 12 and May 14, respectively.

^*^Significant at the 0.05 level.

^**^Significant at the 0.01 level.

In both seasons, significant interaction between hybrid maturity and planting date for grain weight per ear (GWPE) was observed (Table 5). In 2009, JD27 exhibited lower GWPE than XY335 and ZD958 at D1, and ZD958 produced lower GWPE than XY335 and JD27 at D2. GWPE of D2 was significantly greater than D1 for both JD27 and XY335. Conversely, GWPE of D2 was significantly lower than that of D1 for ZD958; In 2010, ZD958 and JD27 exhibited the highest and least GWPE at D1. At D2, XY335 and ZD958 exhibited the highest and least GWPE. GWPE of D2 was significantly greater than D1 for both JD27 and XY335. Conversely, D2 had the significantly lower GWPE than D1 for ZD958.

Significant interaction between hybrid maturity and planting date for number of grains per ear (NGPE) were found in both seasons (Table 5). For planting date effect, ZD958 in 2009 and 2010, and JD27 in 2010 exhibited significant negative and positive change by delayed planting; For hybrid maturity effect, JD27 significant decreased NGPE compared to the other hybrids at D1 in both years. XY335 significant increased NGPE compared to ZD958 at D2 in both years.

Generally, effects of hybrid maturity and planting date on 100-grain weight were weaker compared with parameters mentioned above. No significant difference of 100-grain weight between planting date treatments was found. According to 100-grain weight across hybrids, JD27 had significantly greater 100-grain weight than the other hybrids at D2 in 2009, and also greater XY335 at D1 in 2010 (Table 5).

### Number of grains m^−2^, and grain volume m^−2^

In both years, significant interactions between hybrid maturity and planting date for Number of grains m^-2^ (NGM) were observed (Table 6). For planting date effect, the NGM for ZD958 in 2009 and 2010, and JD27 in 2010 exhibited significant negative and positive change by delayed planting; For hybrid maturity effect, JD27 significant decreased NGM compared to XY335 at D1 in both years. At D2, XY335 significant increased NGM compared to JD27 in 2009, and compared to ZD958 in 2010.

**Table 6:** Effects of planting date and hybrid maturity on number of grains m^−2^, and volume of grains m^−2^ of dryland corn at Lishu city, Northeast China during 2009 and 2010 growing seasons.

	2009			2010		
	JD27	XY335	ZD958	JD27	XY335	ZD958
Planting date	Number of grains m^−2^					
D1^c^	2786Bb	3287Aa	3222ABa	2106B	2908A	3033A
D2^d^	2946B	3424A	2812B	2673ab	2857a	2273b
Difference	160NS	137NS	410*	568*	51NS	761**
	Volume of grains m^−2^ cm^3^ m^−2^					
D1	776b	791ab	801a	613C	707B	809A
D2	831B	865A	668C	686B	768A	601C
Difference	55**	74**	133**	73**	61**	208**

^a^Means within a row followed by a different lower-case letter differ at *P* < 0.05. Means within a row followed by a different upper-case letter differ at *P* < 0.01.

^b^NS, not significant.

^c^D1 represents the planting date of maize in early May. The planting dates for 2009 and 2010 were May 2 and May 4, respectively.

^d^D2 represents the planting date of maize in mid-May. The planting dates for 2009 and 2010 were May 12 and May 14, respectively.

^*^Significant at the 0.05 level.

^**^Significant at the 0.01 level.

Significant interaction between hybrid maturity and planting date treatment for volume of grains m^−2^ (VGM) was found in both seasons (Table 6). For planting date effect, the VGM for JD27 and XY335 in both years exhibited significant positive change by delayed planting. Conversely, the VGM for ZD958 in both years exhibited significant negative change; For hybrid maturity effect, ZD958 significant increased VGM compared to JD27 at D1 in 2009, and compared to the other two hybrids in 2010. At D2, significant difference across three hybrids were found with the order of VGM: XY335>JD2 >ZD958. The results indicated that differences in VGM among treatments were consistent with the differences in grain yield among treatments.

## Discussion

The evaluated hybrids showed different duration from emergence to silking and silking to physiological maturity stages. Short season hybrid JD27 exhibited both lower duration from emergence to silking and silking to physiological maturity stage compared to mid- and full-season hybrids. However, the duration from emergence to silking stage between mid- and full-season hybrids was similar. Also, the notable difference of duration from silking to physiological maturity stage between them was observed. These results differ from those observed by Major et al.^35^ in Canada, who found that the difference in the hybrid cycle were mainly explained by changes in the duration of the period from emergence to flowering.

The results also showed maize physiological response to delayed planting among hybrids was different. Days of emergence to silking stage for all the hybrids consistently shortened as the planting delayed. For DSPM, the short-season hybrid showed numerically difference between planting date treatments. DSPM of the mid-season hybrid became shorter in 2009 and longer in 2010 respectively as the planting delayed. DSPMS of the full-season hybrid showed longer in both years as the planting delayed. The result differs from the observations by Liu et al.^36^. Their data were observed in Wuqiao, Hebei province where the thermal time was plentiful for spring planting maize. They reported that both the durations from emergence to silking and silking to physiological maturity stage for late April planting became longer compared with the mid May planting treatment. Delayed planting effects on flowering and grain maturation of corn also observed by Nielsen et al.^37^ in the eastern U. S. Corn Belt. They found that thermal time from planting to silk emergence decreased an average of 34 GDDs (Growing degree days) for June vs. early May plantings while the grain-fill period decreased an addition 110 GDDs with late planting. They also reported that the three hybrids responded differently to delayed planting with greater GDD decreases occurring with late-maturity hybrids.

Several researchers have investigated the difference in number of leaves among maize hybrids with contrasting maturity. Cao and Wu^38^ indicated that the longer the hybrids cycle length, the greater the number of leaves. However, facing the results we got, the conclusions may be different. In our experiment, the number of leaves was lowest for short-season hybrid with little variations between mid and full-season hybrids. As mentioned above, the duration from emergence to silking was similar between mid- and full-season hybrids. Thus, we concluded that the number of leaves was more closely related to the duration from emergence to silking compared with the hybrid cycle length.

As hypothesized, the longer maturity hybrid usually had grain yield advantage over earlier maturity hybrid when planting at the date of early May, while the earlier maturity hybrid often showed a greater yield than longer maturity hybrid when planting was delayed. This result is supported by Norwood^25^. It reported a full-season hybrid generally produced more grain than a short-season hybrid when planted early and yields of a short-season hybrid were equal to or greater than yields of a full-season hybrid at later planting dates^25^. Our data also exhibited that the highest yield occurring at the treatment combination of the mid-season hybrid and delayed planting date, and the shorter season hybrid typically showed stable and higher grain yield across planting dates.

Grain yield is composed of ears per ha, kernels per ear and kernel weight^39^. Yield also can be expressed as the product of kernels per ha and kernel weight^25^. Numerous researches have consistently demonstrated that grain yield increases were mostly related to an improved number of harvestable kernels per unit land area^39–43^. We investigated the response of maize hybrids with contrasting maturity to planting date in terms of dry matter accumulation and partitioning (biomass yield, harvest index), yield component (ears ha^−1^, kernel ear^−1^, 100-grain weight, number of grains m^−2^, and grain volume m^−2^). The results showed that (i) differences in grain yield among hybrids and planting dates treatment combination was closely related with the differences in biomass yield compared with harvest index. (ii) Grain weight per ear, grain number per ear, and 100-grain weight for short- and mid- season hybrids usually increased as the planting delayed, but for full-season hybrid, most of these yield components decreased following the later planting. (iii) Changes in grain volume per unit of land area among hybrids and planting date treatment combination were consistent with the changes in grain yields, indicating that the yield is determined usually by the sink capacity, this result is consistent with previous investigations^44^. Interestingly, the results also showed that grain volume per unit of land area is more appropriate for measuring the parameter of grain sink capacity compared with the term of grain volume per unit of land area. The finding indicated that difference usually existed in single grain size or/and weight among hybrids. A further study is required to determine the relationship between maize yield and hybrid maturity-planting date interaction for all the Northeast China. Furthermore, it is reported that optimum planting density is usually higher for short-season than for full-season hybrids^28^. For short-season hybrids more plants are needed to reach the same amount of cumulative intercepted radiation^28^ according to their small leaf area per plant and a shorter duration of growth. Thus, more research is needed to evaluate the effects of planting density, hybrids maturity and planting date interactions on maize yield and relative morphophysiological traits.

In addition, in this study,the planting density of each treatment was uniformly set at 67 500 plants per ha, which was mainly based on the following considerations: Yield environment of maize is the determining factor of planting density^45^. Under the yield environment of this experimental site (9–10 t ha^−1^), the maximum yield density of the hybrids XY335 and ZD958 (also used in this study) was 52,500 plants per ha. When the density was increased to 67,500 and 82,500 plants per ha, the yield did not change significantly^44^. Theoretically, the maximum yield density of earlier maturity hybrid of JD27 was higher than those of longer maturity hybrids. Therefore, the density of all treatments was set at 67 500 plants per ha in this experiment.

## Conclusion

(i) Differences in grain yield among hybrids and planting dates treatment combination was closely related with the differences in biomass yield compared with harvest index.
(ii) Grain weight per ear, grain number per ear, and 100-grain weight for short- and mid- season hybrids usually increased as the planting delayed. For full-season hybrid, most of these yield components decreased following the later planting.
(iii) Changes in grain volume per unit of land area among hybrids and planting date treatment combination were consistent with the changes in grain yields, indicating that the yield is determined usually by the sink capacity.

## Materials and methods

### Experimental locations

The experiments were conducted at the Lishu Research Extension Center of CAAS near Siping City(43° 18′ N 124° 18′ E), Jilin province, from 2009 through 2010. The soil was a black loam with a pH of 7.5 and an organic matter content of 26 g kg^−1^. Thirty-year average climatic data for Siping City are annual precipitation of 577.2 mm, mean temperature of 5.8 °C, and a frost-free period of 152 days. The experimental site located in Northeast China, which is a typical spring maize area and is mainly rain fed.

### Management practice

The hybrids were planted by hand at a rate of 67,500 hills ha^−1^, three to four seeds per hill planted and hand-thinned after emergence to 67,500 plants ha^−1^ populations. Herbicides used each year were atrazine [6-chloro-*N*-ethyl-*N*′-(1-methlyethyl)-1,3,5-triazine-2,4-diamine] applied at a rate of 2.2 kg ha^−1^ after planting for weed control. The plots received 150 kg N ha^−1^ before planting and 75 kg N ha^−1^ at 12-leaf stage respectively each year. The soil P level averaged about 14.6 mg·kg^-1^, but 150 kg ha^−1^ P_2_O_5_ was applied, and 150 kg ha^−1^ K_2_O also was applied at the beginning of the study to eliminate any potential deficiencies.

### Experimental design and data collection

The experimental design was a split plot with three replications. Planting date was the main plot, hybrid was the subplot. Each subplot was 4.8 m wide (eight 60-cm rows) by 6 m long. Row 1–2 and 7–8 on each subplot provided a border (four rows) to minimize the impact of adjacent treatments. Maize was planted in early May (D1, the planting dates in 2009 and 2010 were May 2 and May 4, respectively) and mid-May (D2, the planting dates in 2009 and 2010 were May 12 and May 14, respectively) of each year. Three maize hybrids differing in relative maturities were planted in both of year. Hybrids were Jidan27 (JD27), Xianyu335 (XY335), and Zhengdan958 (ZD958), which had maturities of 109 days, 120 days, and 126 days according to recorded calendar time from crop emergence to kernel black layer formation, respectively. The full-season hybrid ZD958 was selected based on their adaptation to the area. The mid-season hybrid XY335 have been successfully grown under dryland conditions in the area. The short-season hybrid JD27 have been widely grown in northern part of Jinlin and southern part of Heilongjiang and was selected to represent hybrids with earlier relative maturities.

Silking date was measured to determine the traits of days from emergence to silking, silking to physiological maturity, and emergence to physiological maturity, while silking date was recorded when 60% of the ears showed silk emergence.

Number of leaves per plant was measured by the following method: Plants from the center four rows of each plot were marked by painting the sixth-leaf red at 8-leaf stage, and accordingly, painting the 12th-leaf red at 14th-leaf stage, and finally to count the leaves of each plant.

Aboveground dry matter, grain yield, and number of ears m^−2^ were determined at physiological maturity by hand harvesting all the plants from the center two rows of each plot, excluding the most exterior plants of each row, comprising an area of 5.93m^2^. Grain moisture percentage was recorded and expressed as kernel water content at harvest stage, and grain yield was weighed and expressed on 14% moisture content basis. Kernel density was calculated from (100-kernel weight × 1000)/100-kernel volume, and 100-kernel weigh and 100-kernel volume (determined by the drainage methods) was recorded as the mean of 3 × 100 kernel random samples. The number of grains per unit surface area (number of grains m^−2^) and per plant were calculated as quotient between grain yield and individual grain weight, both on a 14% moisture content basis. The volume of grains m^-2^ was calculated from (number of grains m^−2^ × 100-kernel volume/100). Harvest index was calculated as the ratio between grain yield and total aboveground biomass at physiological maturity.

Statistical analysis was by PROC ANOVA with mean separation by Fisher's protected LSD and by the linear forward selection component of PROC REG.
